# Synthesis, Characterization, and Anti-Cancer Activity of Some New *N*′-(2-Oxoindolin-3-ylidene)-2-propylpentane hydrazide-hydrazones Derivatives

**DOI:** 10.3390/molecules200814638

**Published:** 2015-08-13

**Authors:** Ayman El-Faham, Muhammad Farooq, Sherine N. Khattab, Nael Abutaha, Mohammad A. Wadaan, Hazem A. Ghabbour, Hoong-Kun Fun

**Affiliations:** 1 Department of Chemistry, College of Science, King Saud University, P.O. Box 2455, Riyadh 11451, Saudi Arabia; 2 Department of Chemistry, Faculty of Science, Alexandria University, P.O. Box 426, Alexandria 21321, Egypt; E-Mail: Sh.n.Khattab@gmail.com; 3 Department of Zoology, College of Science, King Saud University, P.O. Box 2455, Riyadh 11451, Saudi Arabia; E-Mails: nabutaha@ksu.edu.sa (N.A.); Wadaan@ksu.edu.sa (M.A.W.); 4 Department of Pharmaceutical Chemistry, College of Pharmacy, King Saud University, P.O. Box 2457, Riyadh 11451, Saudi Arabia; E-Mails: ghabbourh@yahoo.com (H.A.G.); hfun.c@ksu.edu.sa (H.-K.F.); 5 X-ray Crystallography Unit, School of Physics, Universiti Sains Malaysia, Penang 11800, Malaysia

**Keywords:** valproic acid, isatin, hydrazide-hydrazone, anti-cancer activity

## Abstract

Eight novel *N*′-(2-oxoindolin-3-ylidene)-2-propylpentane hydrazide-hydrazone derivatives **4a**–**h** were synthesized and fully characterized by IR, NMR (^1^H-NMR and ^13^C-NMR), elemental analysis, and X-ray crystallography. The cyto-toxicity and *in vitro* anti-cancer evaluation of the prepared compounds have been assessed against two different human tumour cell lines including human liver (HepG2) and leukaemia (Jurkat), as well as in normal cell lines derived from human embryonic kidney (HEK293) using MTT assay. The compounds **3e**, **3f**, **4a**, **4c**, and **4e** revealed promising anti-cancer activities in tested human tumour cells lines (IC_50_ values between 3 and 7 μM) as compared to the known anti-cancer drug 5-Fluorouracil (IC_50_ 32–50 μM). Among the tested compounds, **4a** showed specificity against leukaemia (Jurkat) cells, with an IC_50_ value of 3.14 μM, but this compound was inactive in liver cancer and normal cell lines.

## 1. Introduction

Hydrazide-hydrazone derivatives are molecules containing a highly reactive group (CO-NH-N=CH) and considered to be a good candidate for development of a new drug [1]. Recently, hydrazide-hydrazones have been considered to be of great interest in medicinal chemistry due to their diverse biological properties, including anti-microbial [2,3,4], anti-mycobacterial [5,6], anti-convulsant [7], analgesic [8], anti-inflammatory [9], anti-platelet [10], anti-tubercular [10,11,12,13], and anti-tumoral activities [14,15,16,17,18,19]. In addition, hydrazide-hydrazones have been reported to elicit anti-cancer [18,19,20,21] and anti-HIV properties [22] and they are therefore increasingly considered to be of great value in medicinal chemistry [16,23,24,25,26,27].

Because of the remarkable biological value of isatin as an important constituent of bioactive compounds exhibiting caspase inhibitory [28,29], antibacterial, and antiproliferative activity [30], Schiff bases of isatin derivatives have antismallpox [31] and GAL3 receptor antagonist capabilities [32]. In addition, the analogous of isatin derivatives displayed inhibitory activity against eLF2 kinase activator [33], TNF-α, CDK2 [34] and SARS protease [35]. Isatin also displays antiviral [36], anti-inflammatory, analgesic [37], and anticonvulsant activities [38].

Valproic acid (VPA, **1**) has an important biological value as a potent antiepileptic molecule [39,40,41], as well as showing inhibition of angiogenesis both *in vitro* and *in vivo* [42,43,44,45]. VPA also acts as a powerful histone deacetylase inhibitor [46,47] and induces differentiation and apoptosis in a variety of malignant cells *in vitro* [48]. Clinical trials with VPA have focused on acute myeloid leukaemia and the myelodysplastic syndromes. When it was used as mono therapy or in combination with all-trans retinoic acid, which synergizes *in vitro*, VPA achieved hematologic improvement in a subset of patients [48].

**Figure 1:**
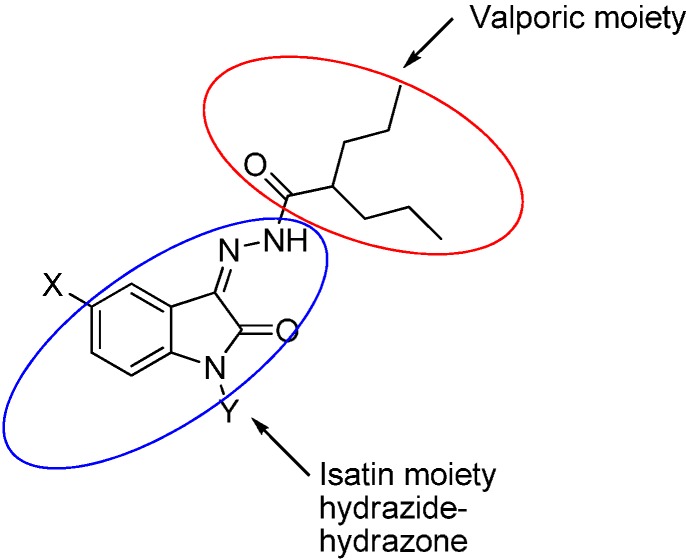
Structure of the target product.

As a continuation to our previously reported data [49,50,51], herein we designed eight isatin hydrazide-hydrazone derivatives, considering some of the factors responsible for such activity, including (i) the presence of isatin moiety; (ii) the presence of the hydrazide-hydrazone functionality; and (iii) valproic acid moiety (Figure 1).

All the prepared compounds were assessed against two different human tumour cell lines, including human liver (HepG2) and leukaemia (Jurkat), as well as in normal cell lines derived from human embryonic kidney (HEK293) using MTT assay.

## 2. Results and Discussion

### 2.1. Chemistry

**Scheme 1:**
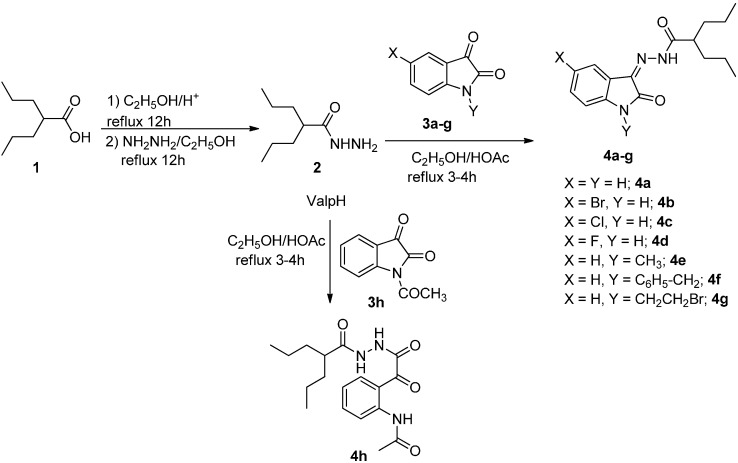
Synthesis of *N*′-(2-oxoindolin-3-ylidene)-2-propylpentanehydrazide Derivatives.

Compound **2** was prepared following the reported method [49] and condensed with isatin derivatives **3a**–**h** in the presence of 2–3 drops of glacial HOAc and ethanol as the solvent to afford products **4a**–**h** (Scheme 1). The structures of all synthesized compounds were in a good agreement with their spectral data.

The IR spectra of the compounds **4a**–**g** reveal absorption bands in the region 3195–3217 cm^−1^ corresponding to the (NH), a band at 1720 and 1688 cm^−1^ corresponding to the C=O group, and a peak around 1596 cm^−1^ related to the C=N bond. The NMR of all the products **4a**–**g** are in good agreement with their structures (Figures S1–S7).

As a prototype ^1^H-NMR of **4g** showed multiple peaks at δ 0.86–0.89, 1.27–1.29, 1.40–1.44, 1.56–1.60 ppm, and a broad singlet at δ 2.51 ppm corresponding to the valporic acid moiety (2 CH_3_, 4 CH_2_, and CH, respectively). Also, two triplet peaks were observed at δ 3.77 and 4.20 ppm corresponding to the two methylene group (CH_2_-CH_2_Br), respectively. The observed peaks in the aromatic region at δ 7.18 (t, 1H, Ar-H), 7.32 (d, 1H, Ar-H), 7.53 (t, 1H, Ar-H), and 7.88 (brs, 1H, ArH) are related to the isatin moiety, while the broad singlet peak at δ 12.28 ppm is related to the NH. The ^13^C-NMR of **4g** showed peaks at δ 14.5, 20.6, 29.9, 35.3, 39.7, 42.2, and 46.5 ppm, corresponding to the valporic acid moiety and the two methylene groups, in addition to seven peaks related to the aromatic carbons and imino function group. The two peaks at δ 169.8 and 176.0 ppm corresponded to the carbonyl groups of the isatin moiety and the hydrazide group, respectively.

**Figure 2:**
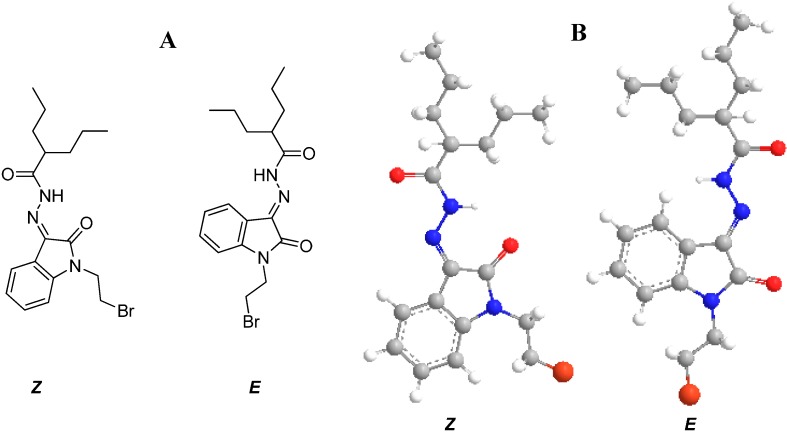
(**A**) The expected geometrical isomers, *Z* and *E* forms, of *N*′-(1-(2-bromoethyl)-2-oxoindolin-3-ylidene)-2-propylpentane hydrazide; (**B**) The expected geometrical isomers, the 3D structure of *Z* and *E* forms, of *N*′-(1-(2-bromoethyl)-2-oxoindolin-3-ylidene)-2-propyl pentane hydrazide.

It is expected that compound **4g** could adopt two different geometrical isomers (*Z* and *E*) as shown in Figure 2A. Therefore, it is considered worthwhile to model the compounds using molecular mechanics MM2 calculations. In addition, quantum chemical calculations were carried out with the GAUSSIAN 98 suite of programs. Geometry optimizations were carried out using the DFT level (B3LYP/6-31G **) of theory to assess the relative stability of the *Z* and *E* isomeric species. Calculated relative energies of **4g** *Z* and *E* isomers are −3585.4860873 au and 3585.4635100 au, respectively. Computed energies indicate the stability of the *Z* isomer over the *E* one by 0.0225773 au (14.1675 kcal/mol) (Figure 2B).

**Figure 3:**
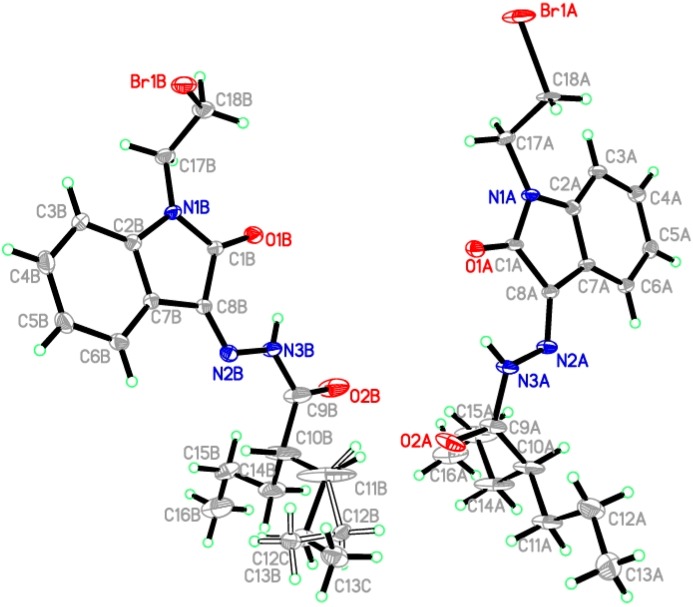
ORTEP diagram of the asymmetric unit consisting of two symmetry-independent molecules. The ellipsoids are drawn at the 50% probability level with hydrogen atoms being shown as spheres of arbitrary radii. One of the molecules has disorder in the side chain C11B—C13B.

The X-ray single crystal structure determination of compound **4g** (CCDC: 996592) confirmed that the structure exists in the *Z*-conformer rather than the *E*-conformer (Figure 3). The details of data collection and structure refinement are listed in Table 1, and the X-ray structures are shown in Figure 3. Compound **4g** crystallized from ethanol in the triclinic space group *P*-1. All bond lengths and angles are in normal ranges [52].

**Figure 4:**
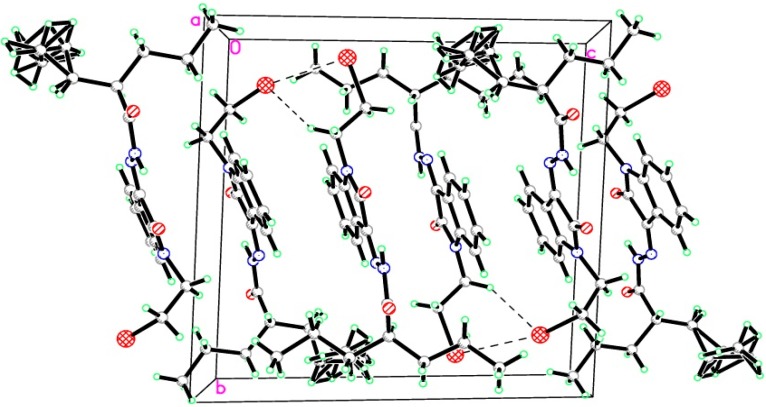
Crystal packing showing intermolecular hydrogen bonds as dashed lines.

In the crystal packing (Figure 4), the molecules are linked by intermolecular C17A—H17B···Br1B, C17B—H17D···O2B and C18A—H18B···O2A hydrogen bonds (Table 2) into two-dimensional networks parallel to the bc plane (Figure 4 and Table 2). The asymmetric unit contains two molecules of the compound with disorder in one of the ethylene arms. The molecular structure of compound **4g** is composed of a 2-oxoindoline ring (C1/N1/C2-C8) which is linked with two side chains at N1 and C8. The two molecules in the asymmetric unit are with Z configuration about the C8=N2 double bond (Figure 3). The Z configuration of **4a** is stabilized with intramolecular hydrogen N3—H3N···O2 (Table 2). The single bond N2—N3 is clearly characterized by the distances of 1.355 (3) Å and 1.361 (4) Å, respectively, for molecules A and B. The double bond of C8=N2 is characterized by the distances of 1.298 (3) Å and 1.283 (4) Å, for molecules A and B, respectively.

**Table 1:** Details of data collection and structure refinement for compound **4g**.

Compound	4g
Empirical formula	C_18_H_24_BrN_3_O_2_
Formula weight	349.21
Temperature (K)	100
Wavelength (A)	Mo Kα radiation, λ = 0.71073 Å
Crystal system, Space group	Triclinic, *P*-1
Crystal	Plate, yellow
*a* (Å)	8.8126 (11)
*b* (Å)	14.4104 (18)
*c* (Å)	14.479 (2)
α (°)	92.002 (4)
β (°)	97.498 (4)
γ (°)	92.598 (4)
Volume (Å^3^)	1819.6 (4)
Z, D_calc_ (Mg·m^−3^)	4, 1.275
*F* (000)	816
Crystal size (mm)	0.58 × 0.41 × 0.12
θ Range for data (°)	2.33–28.11
Collection Limiting indices	−12 ≤ h ≤ 12, −20 ≤ k ≤ 20, −20 ≤ k ≤ 20
Reflections collected/unique	81,300/6824 [R_int_ = 0.136]
restraints/parameters	112/469
Goodness-of-fit on *F*^2^	1.009
Final R indices[I > 2s(I)]	R1 = 0.053, wR2 = 0.135
Absorption correction	Multi-scan, SADABS V2012/1 (Bruker AXS Inc., Madison, WI, USA)
Instrument	Bruker APEX-II D8 venture diffractometer

**Table 2:** Hydrogen-bond geometry (Å, °).

*D*—H···*A*	*D*—H	H···*A*	*D*···*A*	*D*—H···*A*
N3B−H3NB···O1B	0.81 (3)	2.10 (3)	2.779 (3)	141 (3)
N3A−H3NA···O1A	0.86 (3)	2.11 (3)	2.799 (3)	138 (3)
C10A−H10A···N2A	0.9800	2.3700	2.815 (4)	107.00
C10B−H10B···N2B	0.9800	2.3600	2.833 (4)	109.00
C17A−H17A···O1A	0.9700	2.5700	2.930 (3)	102.00
C17A−H17B···Br1B ^i^	0.9700	2.9100	3.779 (3)	149.00
C17B−H17D···O2B ^ii^	0.9700	2.4100	3.139 (4)	131.00
C18A−H18B···O2A ^iii^	0.9700	2.2900	3.096 (3)	140.00

Symmetry codes: (^i^) *x* − 1, *y*, *z*; (^ii^) −*x* + 1, −*y* + 1, −*z*; (^iii^) −*x* + 1, −*y* + 1, −*z* + 1.

Reaction of valporic acid hydrazide **2** with *N*-acetylisatin **3h** proceeds in a different way, where the ring opening occurred to afford the product **4h**. The IR and NMR data proved its structure in the open form, due to the attack of the hydrazide group on C2 instead of C3 [53]. The IR spectrum of **4h** reveals absorption bands in the region 3310 and 3223 cm^−1^ corresponding to the NH, a band at 1720, 1709, 1688, and 1608 cm^−1^ corresponding to the C=O and C=N group, respectively. ^1^H-NMR of **4h** showed multiple peaks at δ 0.86–0.89, 1.27–1.29, 1.40–1.44, 1.56–1.60, and a broad singlet peak at δ 2.21 ppm corresponding to the valporic acid moiety (2 CH_3_, 2 CH_2_, and 2CH_2_, CH, respectively). The observed peak at δ 2.16 ppm is corresponding to the *N-*acetyl group. The observed peaks in the aromatic region at δ 7.28 (t, 1H), 7.68 (t, 1H), 7.99 (d, 1H), and 8.17(d, 1H) ppm, are related to the phenyl ring, while the two singlet peaks at δ 10.03 and 10.72 ppm corresponded to the two NH. The ^13^C-NMR of **4h** showed peaks at δ 14.6, 20.6, 29.9, 35.3, 43.8, and 46.5 ppm corresponding to the valporic acid moiety group, while the acetyl group was observed at δ 25.0 ppm, in addition to six peaks related to the aromatic carbons at δ 121.5, 121.8, 123.8, 133.6, 135.9, and 140.3 ppm. The three peaks observed at δ 164.1, 169.6, and 174.7 ppm related to the three CONH groups, while the peak observed at δ 193.0 ppm is related to the α-ketoamide group.

### 2.2. Anti-Cancer Activity

The cyto-toxicity activity was measured *in vitro* in HepG2, Jurkat, and HEK 293 cells using the MTT 1-(4,5-dimethylthiazol-2-yl)-3,5-diphenylformazan colorimetric assay. For comparison, 5-Fluorouracil (5-FU) was used as a standard anti-cancer drug. Treatment with dimethylsulfoxide (DMSO) was used as a control for the cancer cells.

**Figure 5:**
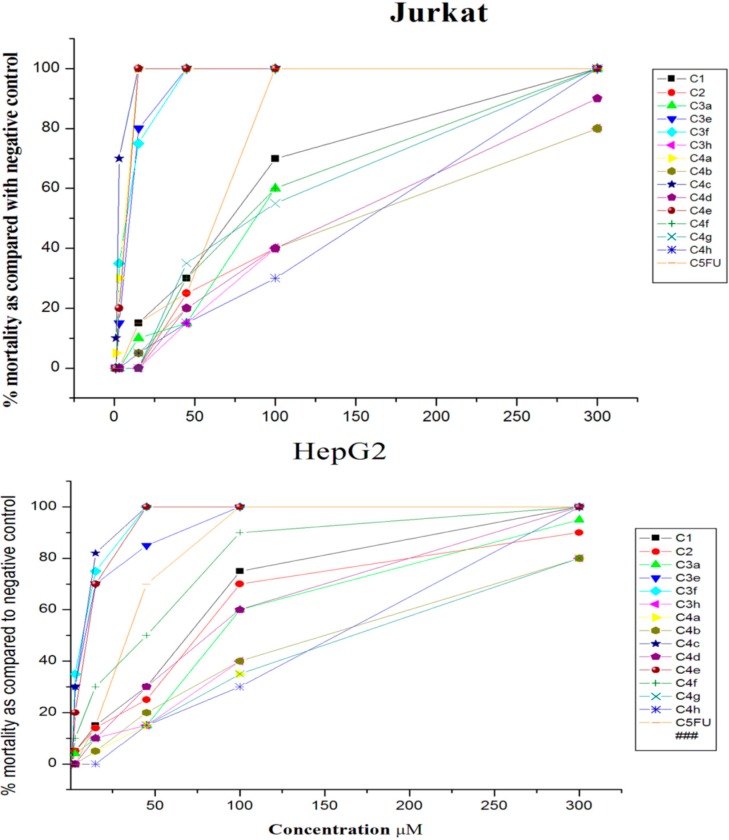

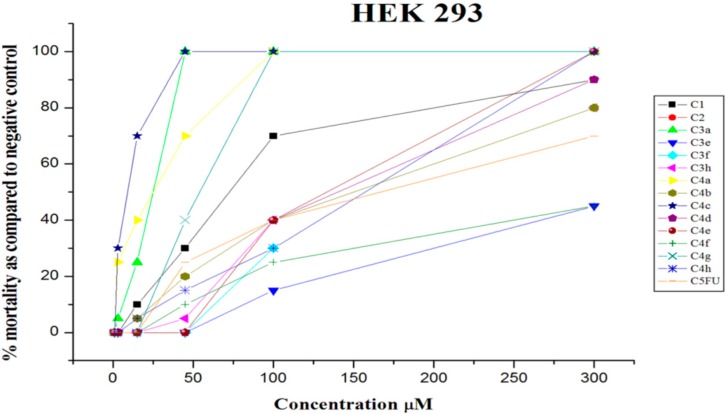
Dose-dependent responses of hydrazide-hydrazone derivatives in three cell lines. Each line represents the mean of three different experiments representing the % of mortality as compared to mock treated control cells.

The anti-cancer activity of the new prepared compounds against the HepG2 cell line revealed that compounds **3a**, **3h**, **4a**, **4b**, **4f**, **4g**, and **4h** showed no anti-cancer activity. Similarly, the starting compounds **1** and **2** did not show promising anti-cancer activity in HepG2 cells. Compound **3e** exhibited higher potency against HepG2 cell line with IC_50_ = 2.82 ± 40.7 μM, which is much lower than the IC_50_ value of 5-FU (32.15 ± 48.1 μM). Moreover, the results showed that compounds **3f**, **4c**, and **4e** were also found to be potent and selective against HepG2 with IC_50_ values of 3.13 ± 42.0, 4.39 ± 43.2, and 7.61 ± 44.6 μM, respectively, which are also much lower than 5-FU (Figure 5 and Table 3).

The anti-cancer profile of newly synthesized compounds was also tested against leukaemia cells (Jurkat cells, originated from human T lymphocytes). The compound **4c** was most active, with an IC_50_ value of just 2.90 ± 36.3 μM, which was much lower than the positive control (5-FU IC_50_ = 51.16 ± 47.4 μM). Similarly, the compounds **3f**, **4a**, and **4c** showed strong activity and IC_50_ values were much lower as compared to 5-FU (Table 3 and Figure 5). The order of activity was **3f**, **4a**, **4e**, and **3e** in ascending order (Table 3 and Figure 5). The compound **1** showed a weaker level of activity against Jurkat cells (IC_50_ 79.35 ± 40.7 μM) and, indeed, this was much weaker than 5-FU (IC_50_ 51.16 ± 47.4 μM).

**Table 3:** *In vitro* cytotoxicity activity of the synthesized compounds in two cancer and one normal cell line(s) as measured with MTT assay *.

Compd. No.	IC_50_ (μM)		
	HepG2	Jurkat	HEK293
**1**	69.74 ± 40.8	79.35 ± 40.7	62.98 ± 38.2
**2**	68.50 ± 37.1	N.A.	N.A.
**3a**	N.A.	N.A.	16.73 ± 50.0
**3e**	2.82 ± 40.7	7.13 ± 46.0	N.A.
**3f**	3.13 ± 42.0	3.13 ± 42.0	N.A.
**3h**	N.A.	N.A.	N.A.
**4a**	N.A.	3.15 ± 43.3	N.A.
**4b**	N.A.	N.A.	26.08 ± 31.3
**4c**	4.39 ± 43.2	2.90 ± 36.3	4.77 ± 42.7
**4d**	N.A.	N.A.	N.A.
**4e**	7.61 ± 44.6	3.19 ± 46.9	N.A.
**4f**	N.A.	N.A.	N.A.
**4g**	N.A.	N.A.	45.43 ± 48.9
**4h**	N.A.	N.A.	N.A.
**5-FU**	32.15 ± 48.1	51.16 ± 47.4	N.A.
**DMSO**	N.A.	N.A.	N.A.

* Data were expressed as the mean ± standard deviation (SD) of six independent experiments; N.A.: IC_50_ values more than 100 μM was considered as No Activity.

Finally the cyto-toxicities of these compounds were screened in the normal human embryonic kidney (HEK293) cell line, in order to assess whether these compounds are active only in cancer cells or whether they possess toxicity against normal cells as well. The results revealed that the compound **4c** turned out to be most toxic by disrupting the cell survival of normal cells (HEK 293) with IC_50_ 4.77 ± 42.7 μM. The compounds **3a**, **4b**, and **4g** also possessed some level of cyto-toxicity against HEK 293 cells with IC_50_ values 16.73 ± 50.0, 26.08 ± 31.3, and 45.43 ± 48.9 μM, respectively. The IC_50_ values of these compounds in HEK cells, however, are much higher compared to their activity in cancer cells (Table 3 and Figure 5). The compounds **3e**, **3f**, **4a**, and **4e** showed no activity in HEK 293 cells, but strong activity in liver (HepG2) and leukaemia (Jurkat) cancer cells, suggesting that these compounds are potent and potentially viable anti-cancer molecules.

## 3. Experimental Section

### 3.1. Chemistry

#### 3.1.1. Materials

The solvents used were of HPLC reagent grade. Melting points were determined with Melting points were obtained in open capillary tubes using a MEL-Temp melting point apparatus (Sigma-Aldrich Chemie GmbH, 82024 Taufkirchen, Germany) and are uncorrected and are uncorrected. Infrared (IR) spectra were recorded on a Perkin-Elmer 1600 series Fourier transform instrument (PerkinElmer Life and Analytical Sciences, Shelton, CT, USA) as KBr pellets. Nuclear Magnetic resonance spectra (^1^H-NMR and ^13^C-NMR spectra) were recorded on 400 MHz JEOL spectrometer (JEOL Ltd., Tokyo, Japan) at room temperature. Chemical shifts are reported in parts per million (ppm) and are referenced relative to residual solvent (e.g., CHCl_3_ at δ 7.26 ppm for CDCl_3_, DMSO at δ 2.50 ppm for DMSO-*d*_6_). Spin multiplicities are represented by the following signals: singlet (s), broad singlet (br s), doublet (d), broad doublet (br d), doublet of doublets (dd), triplet (t), doublet of triplets (dt), and multiplet (m). Elemental analyses were performed on a Perkin-Elmer 2400 elemental analyzer (PerkinElmer Inc., Waltham, MA USA), and the values found were within ±0.3% of the theoretical values. Follow-up of the reactions and checks of the purity of the compounds was done by TLC on silica gel-protected aluminum sheets (Type 60 GF254, Merck Millipore, Billerica, MA, USA) and the spots were detected by exposure to UV-lamp (Company Seven, Montpelier, MD, USA) at λ 254 nm for a few seconds. The compounds were named using Chem. Draw Ultra version 11, Cambridge soft Corporation. Crystallographic data for the structure reported in this paper have been deposited at the Cambridge Crystallographic Data Center and allocated with the deposition numbers: CCDC 996592 for compound **4g**. CCDC 996592 contains the supplementary crystallographic data for this paper. These data can be obtained free of charge via http://www.ccdc.cam.ac.uk/conts/retrieving.html (or from the CCDC, 12 Union Road, Cambridge CB2 1EZ, UK; Fax: +44 1223 336033; E-mail: deposit@ccdc.cam.ac.uk).

#### 3.1.2. Synthesis of Valproic Hydrazide **2**


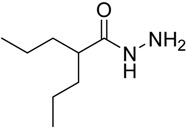


The product was prepared according to the reported procedure [50] and obtained as white needle crystals, in 85%, yield; mp 123–124 °C; IR (KBr): 3284 (NH), 1631 (CO, amide) cm^−1^; ^1^H-NMR (CDCl_3_) δ (ppm): 0.86 (t, *J* = 4.4 Hz, 6H, 2CH_3_), 1.14–1.39 (m, 6H, 3CH_2_), 1.53–1.604 (m, 2H, CH_2_), 1.95–2.01 (m, 1H, CH), 3.95 (brs, 2H, NH_2_), 7.05 (s, 1H, NH). ^13^C-NMR (CDCl_3_) δ (ppm): 14.11, 20.86, 35.05, 45.61, 177.06.

#### 3.1.3. Synthesis of Compounds **3e**–**h**

Compounds **3e**–**h** were prepared following the reported procedure [54]. The entire prepared compounds were in a good agreement with the reported data.

#### 3.1.4. General Method for Preparation of *N*′-(2-Oxoindolin-3-ylidene)-2-propylpentane Hydrazide Derivatives **4a**–**h**

A solution of valproic hydrazide **2** (316 mg, 2 mmol) in ethanol (20 mL) was added to a solution of substituted isatin **3a**–**h** (1 mmol) in ethanol (20 mL), and glacial acetic acid (2 drops); the reaction mixture was refluxed for 3–4 h. The product was separated out on cooling, filtered, and recrystallized from ethanol or ethylacetate to afford *N*′-(2-oxoindolin-3-ylidene)-2-propylpentanehydrazide derivatives **4a**–**h**.

(*Z*) *N*′-(2-Oxoindolin-3-ylidene)-2-propylpentane Hydrazide **4a**


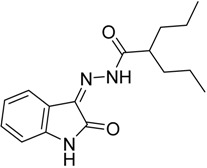


The product was obtained as yellow solid, in 89% yield; mp 175–176 °C. IR (KBr): 3194 (NH), 1720 (CO), 1663 (C=N), 1602 (CO) cm^−1^; ^1^H-NMR (DMSO-*d*_6_) δ (ppm): 0.85-0.86 (m, 6H, 2 CH_3_), 1.26–1.27 (m, 4H, 2 CH_2_), 1.40–1.42 (m, 2H, CH_2_), 1.56–1.58 (m, 2H, CH_2_), 2.51 (brs, 1H, CH), 6.90 (d, *J* = 4.6 Hz, 1H, Ar-H), 7.06 (brs, 1H, Ar-H), 7.38 (brs, 1H, ArH), 8.02 (brs,1H, ArH), 10.80 (s, 1H, NH), 11.08 (s, 1H, NH); ^13^C-NMR (DMSO-*d*_6_) δ (ppm): 14.5, 20.6, 35.1, 39.8,111.2, 115.9, 122.2, 126.6, 133.1, 144.3, 165.3, 169.80, 172.5. *Anal.* Calcd for C_16_H_21_N_3_O_2_: C, 66.88; H, 7.37; N, 14.62; found: C, 66.70; H, 7.44; N, 14.78.

(*Z*) *N*′-(5-Bromo-2-oxoindolin-3-ylidene)-2-propylpentane Hydrazide **4b**


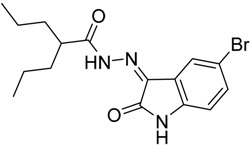


The product was obtained as yellow solid, in 86% yield; mp 172–173 °C; IR (KBr): 3222 (NH), 1729 (CO), 1663 (C=N), 1609 (CO) cm^−1^; ^1^H-NMR (DMSO-*d*_6_): δ (ppm): 0.85–0.89 (m, 6H, 2 CH_3_), 1.27–1.29 (m, 4H, 2 CH_2_), 1.40–1.43 (m, 2H, CH_2_), 1.57–1.59 (m, 2H, CH_2_), 2.50 (brs, 1H, CH), 6.90 (brs, 1H, Ar-H), 7.54 (brs, 1H, ArH), 8.02 (brs, 1H, ArH), 10.91 (s, 1H, NH), 11.31 (s, 1H, NH); ^13^C-NMR (DMSO-*d*_6_) δ (ppm): 14.6, 20.6, 35.1, 39.8, 112.9, 117.5, 122.2, 128.7, 135.1, 144.3, 166.3, 170.80, 172.5. *Anal.* Calcd for C_16_H_20_BrN_3_O_2_: C, 52.47; H, 5.50; N, 11.47; found: C, 52.66; H, 5.61; N, 11.23.

(*Z*) *N*′-(5-Chloro-2-oxoindolin-3-ylidene)-2-propylpentane Hydrazide **4c**


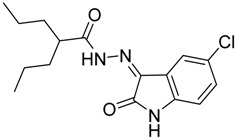


The product was obtained as yellow solid, in 88% yield; mp 154–156 °C; IR (KBr): 3233 (NH), 1720 (CO), 1689 (C=N), 1620 (CO) cm^−1^; ^1^H-NMR (DMSO-*d*_6_) δ (ppm): 0.87–0.93 (m, 6H, 2 CH_3_), 1.26–1.29 (m, 4H, 2 CH_2_), 1.40–1.43 (m, 2H, CH_2_), 1.57–1.59 (m, 2H, CH_2_), 2.51 (brs, 1H, CH), 6.91 (m, 1H, Ar-H), 7.42 (m, 1H, ArH), 8.2 (brs, 1H, ArH), 10.91 (s, 1H, NH), 11.35 (brs, 1H, NH); ^13^C-NMR (DMSO-*d*_6_) δ (ppm): 14.6, 20.6, 35.3, 39.7, 112.8, 118.5, 122.0, 126.2, 134.1, 145.3, 165.3, 170.80, 172.5. *Anal.* Calcd for C_16_H_20_ClN_3_O_2_: C, 59.72; H, 6.26; N, 13.06; found: C, 59.55; H, 6.18; N, 13.33.

(*Z*) *N*′-(5-Fluoro-2-oxoindolin-3-ylidene)-2-propylpentane Hydrazide **4d**


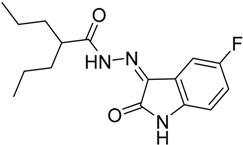


The product was obtained as yellow solid in 89% yield; mp 180–182 °C; IR (KBr): 3181 (NH), 1721 (CO), 1686 (C=N), 1630 (CO) cm^−1^; ^1^H-NMR (DMSO-*d*_6_) δ (ppm): 0.86–0.89 (m, 6H, 2 CH_3_), 1.26–1.27 (m, 4H, 2 CH_2_), 1.40–1.43 (m, 2H, CH_2_), 1.57–1.59 (m, 2H, CH_2_), 2.50 (brs, 1H, CH), 6.88 (m, 1H, Ar-H), 7.24 (m, 1H, ArH), 8.2 (brd, 1H, ArH), 10.81 (s, 1H, NH), 11.23 (brs, 1H, NH); ^13^C-NMR (DMSO-*d*_6_) δ (ppm): 14.6, 20.6, 35.3, 39.7, 112.8, 118.5, 119.8, 124.2, 134.1, 140.3, 162.3, 169.80, 172.5. *Anal.* Calcd for C_16_H_20_FN_3_O_2_: C, 62.94; H, 6.60; N, 13.76; found: C, 63.13; H, 6.77; N, 13.52.

(*Z*) *N*′-(1-Methyl-2-oxoindolin-3-ylidene)-2-propylpentane Hydrazide **4e**


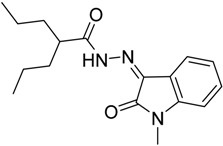


The product was obtained as yellow solid, in 89% yield; mp 154–156 °C; IR (KBr): 1720 (CO), 1689 (C=N), 1620 (CO) cm^−1^; ^1^H-NMR (DMSO-*d*_6_) δ (ppm): 0.85–0.87 (m, 6H, 2 CH_3_), 1.24–1.29 (m, 4H, 2 CH_2_), 1.40–1.43 (m, 2H, CH_2_), 1.56–1.59 (m, 2H, CH_2_), 2.50 (brs, 1H, CH), 3.18 (s, 3H, CH_3_), 7.08–7.14 (m, 2H, Ar-H), 7.46 (m, 1H, ArH), 8.20 (brs, 1H, ArH), 11.23 (brs, 1H, NH); ^13^C-NMR (DMSO-*d*_6_) δ (ppm): 14.5, 20.6, 27.6, 35.3, 39.7, 115.3, 119.6, 122.7, 129.2, 131.1, 134.8, 164.0, 169.80, 171.5. *Anal.* Calcd for C_17_H_23_N_3_O_2_: C, 67.75; H, 7.69; N, 13.94; found: C, 67.63; H, 7.78; N, 14.21.

(*Z*) *N*′-(1-Benzyl-2-oxoindolin-3-ylidene)-2-propylpentane Hydrazide **4f**


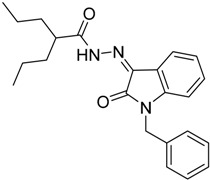


The product was obtained as yellow solid, in 83% yield; mp 170–172 °C; IR (KBr): 1718 (CO), 1686 (C=N), 1620 (CO) cm^−1^; ^1^H-NMR (DMSO-*d*_6_) δ (ppm): 0.86-0.89 (m, 6H, 2 CH_3_), 1.27–1.29 (m, 4H, 2 CH_2_), 1.40–1.44 (m, 2H, CH_2_), 1.56–1.60 (m, 2H, CH_2_), 2.51 (brs, 1H, CH), 4.96 (s, 2H, CH_2_), 7.00–7.20 (m, 2H, Ar-H), 7.33–7.39 (m, 6H, ArH), 8.20 (brs, 1H, ArH), 11.28 (brs, 1H, NH); ^13^C-NMR (DMSO-*d*_6_) δ (ppm): 14.6, 20.6, 35.3, 39.7, 43.2, 115.4, 122.8, 122.9, 126.8, 127.8, 128.1, 129.2, 129.3, 136.8, 144.2, 164.2, 170.5. *Anal.* Calcd for C_23_H_27_N_3_O_2_: C, 73.18; H, 7.21; N, 11.13; found: C, 73.41; H, 7.40; N, 10.94.

(*Z*)-*N*′-(1-(2-Bromoethyl)-2-oxoindolin-3-ylidene)-2-propylpentane Hydrazide **4g**


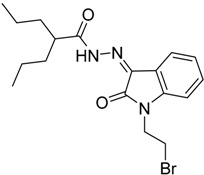


The product was obtained as yellow crystals, in 83% yield; mp 90–91 °C; IR (KBr): 1700 (CO), 1686 (C=N), 1608 (CO) cm^−1^; ^1^H-NMR (DMSO-*d*_6_) δ (ppm): 0.86–0.89 (m, 6H, 2 CH_3_), 1.27–1.29 (m, 4H, 2 CH_2_), 1.40–1.44 (m, 2H, CH_2_), 1.56–1.60 (m, 2H, CH_2_), 2.51 (brs, 1H, CH), 3.77 (t, *J* = 6.8 Hz, 2H, CH_2_-CH_2_Br), 4.20 (t, *J* = 6.4 Hz, 2H, CH_2_-CH_2_Br), 7.18 (t, *J* = 8.4 Hz, 1H, Ar-H), 7.32 (d, *J* = 8.0 Hz, 1H, Ar-H), 7.53 (t, *J* = 8.0 Hz, 1H, Ar-H), 7.88 (brs, 1H, ArH), 12.28 (brs, 1H, NH); ^13^C-NMR (DMSO-*d*_6_) δ (ppm): 14.5, 20.6, 29.9, 35.3, 39.7, 42.2, 46.5, 110.9, 119.7, 122.7, 123.2, 134.1, 142.9, 164.0, 169.80, 176.0. *Anal.* Calcd for C_18_H_24_BrN_3_O_2_: C, 54.83; H, 6.13; N, 10.66; found: C, 55.12; H, 6.23; N, 10.90.

*N*-(2-(2-Oxo-2-(2-(2-propylpentanoyl)hydrazinyl)acetyl)phenyl) Acetamide **4h**


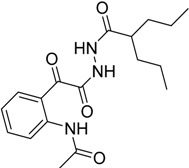


The product was obtained as yellowish white solid, in 83% yield; mp 180–181 °C; IR (KBr): 3310 (NH), 3223(NH), 1720 (CO), 1709 (CO), 1686 (C=N), 1608 (CO) cm^−1^; ^1^H-NMR (DMSO-*d*_6_) δ (ppm): 0.86–0.89 (m, 6H, 2 CH_3_), 1.27–1.29 (m, 4H, 2 CH_2_), 1.40–1.44 (m, 2H, CH_2_), 1.56–1.60 (m, 2H, CH_2_), 2.16 (s, 3H, CH_3_), 2.21 (brs, 1H, CH), 7.28 (t, *J* = 8.0 Hz, 1H, Ar-H), 7.68 (t, *J* = 8.0 Hz, 1H, Ar-H), 7.99 (d, *J* = 7.3 Hz, 1H, Ar-H), 8.17(d, *J* = 8.1 Hz, 1H, ArH), 10.03 (s, 1H, NH), 10.64 (s, 1H, NH), 10.72 (s, 1H, NH);^13^C-NMR (DMSO-*d*_6_) δ (ppm): 14.6, 20.6, 25.0, 29.9, 35.3, 43.8, 46.5, 121.5, 121.8, 123.8, 133.6, 135.9, 140.3, 164.1, 169.6, 174.7, 193.0. *Anal.* Calcd for C_18_H_25_N_3_O_4_: C, 62.23; H, 7.25; N, 12.10; found: C, 62.55; H, 7.42; N, 12.38.

### 3.2. X-ray Crystallography

Single crystals were obtained by slow evaporation from ethanol. A good crystal with a suitable size was selected for X-ray diffraction analysis. Data were collected on a D8 Venture area diffractometer equipped with graphite monochromatic MoK/α radiation (λ = 0.71073 Å) at 100 K. Cell refinement and data reduction were done by Bruker SAINT (Bruker, Madison, WI USA); the program used to solve structure and refine structure is SHELXS-97 [55]. The final refinement was performed by full-matrix least-squares techniques with anisotropic thermal data for non-hydrogen atoms on *F*^2^. All the hydrogen atoms were placed in calculated positions and constrained to ride on their parent atoms. Multi-scan absorption correction was applied by use of SADABS software [56].

### 3.3. Biology

#### 3.3.1. Cell Culture and Cell Viability Assay

The stock concentration of the entire compound in DMSO was 10 mM and this concentration was used to prepare the working dilution. The final DMSO concentration used in the experiments was ≤0.5% as the working concentration. Human liver cancer cell lines (HepG2), human leukemia cell line (Jurkat), and human embryonic kidney cell line (HEK293) cells were cultured in high glucose Dulbecco’s Modified Eagle Medium (DMEM: Life technologies cat #11995073) supplemented with 10% Fetal bovine serum (FBS: Life technologies cat #16000044) in a humidified incubator with 5% CO_2_ at 37 °C. Around 2 ×10^3^ cells were seeded in each well of a 24-well cell culture plate and were allowed to adhere and grow for 24 h. The Jurkat cells grow in suspension so they were cultured and allowed to grow overnight before the addition of compounds. The compounds in serial dilutions (1, 3, 15, 45, 100, and 300 μM) were added after 24 h of culture and the cells were cultured for another 24 h at 37 °C. The cell viability was determined in each experiment using MTT 1-(4,5-dimethylthiazol-2-yl)-3,5-diphenylformazan colorimetric assay. Briefly, the treated or untreated cells were trypsenized and centrifuged, and the resulting pellet was re-suspended in 100 μL of DMEM serum-free medium and incubated at 37 °C for 2 h. After incubation, 20 μL of MTT solution (5 mg/mL in PBS: Sigma Aldrich cat #M2003) was added to each well and further incubated for 2 h. The plate was centrifuged at 40,000 rpm for 10 min then the medium was removed from each well and 2-propanol containing 0.04 M HCl was added to dissolve the formazan produced in the cells. The optical density of the formazan product in solution was measured with a microplate reader at 540 nm. The experiment was conducted in triplicate. Data were calculated as percent of cell viability by the following formula:


% cell viability = (Mean absorbance in test wells/Mean absorbance in control wells) × 100
(1)

The IC_50_ values were calculated from the means of six different concentrations by Bio Data Fit 1.02 using the software Bio Tool Kit (Version 300; Chang Bioscience Inc., Castro Valley, CA, USA) and online program http://ic50.tk/index.html.

#### 3.3.2. Statistics

All data were analyzed using Origin (Version 6.1052; Origin Lab Corp Northampton, MA, USA). One-way ANOVA analysis of variance and student T TEST was used to compare different experimental groups, and data were considered statistically significant for *p* values less than 0.05.

## 4. Conclusions

In conclusion, the screening results from human cancer and normal cell lines suggested that isatin derivatives were remarkably influenced by various substituents on the isatin ring and at the *N*-terminal of the hydrazide-hydrazone moiety. The data revealed that replacement of *N*-hydrogen at position 1 of the isatin moiety **3a** by methyl and benzyl of the targeted compound (**3e** and **3f**) noticeably enhanced the activity against the selected two cell lines, HepG2 and Jurkat, for compound **3f**. The presence of the valproic acid moiety as hydrazide-hydrazone derivatives **4a** make the compound more targeted towards Jurakt cells *in vitro*. Introducing the chlorine atom to the molecule **4c**, meanwhile, increased the reactivity more significantly than with **4a** and **3a** towards the three cell lines HepG2, Jurkat, and HEK293. Furthermore, the methyl group in the same analogous compound **4e** increased the reactivity towards the two cell lines HepG2 and Jurkat more than the benzyl group **4f**. The rest of the compounds had no effect on the three cell lines. Further studies to assess the effect of more derivatives on various cancer cell biomarkers are currently underway in our lab.

## Supplementary Materials

Supplementary materials can be accessed at: http://www.mdpi.com/1420-3049/20/08/14638/s1.
