# Supplementary material for: Synthesis, Characterization, and Anti-Cancer Activity of Some New N′-(2-Oxoindolin-3-ylidene)-2-propylpentane hydrazide-hydrazones Derivatives
Source: Molecules. 2015 Aug 13;20(8):14638–55. doi: 10.3390/molecules200814638 (PMC6332339; doi:10.3390/molecules200814638)
Supplement: Supplementary file 1 [file molecules-20-14638-s001.pdf]

# Supplementary Materials

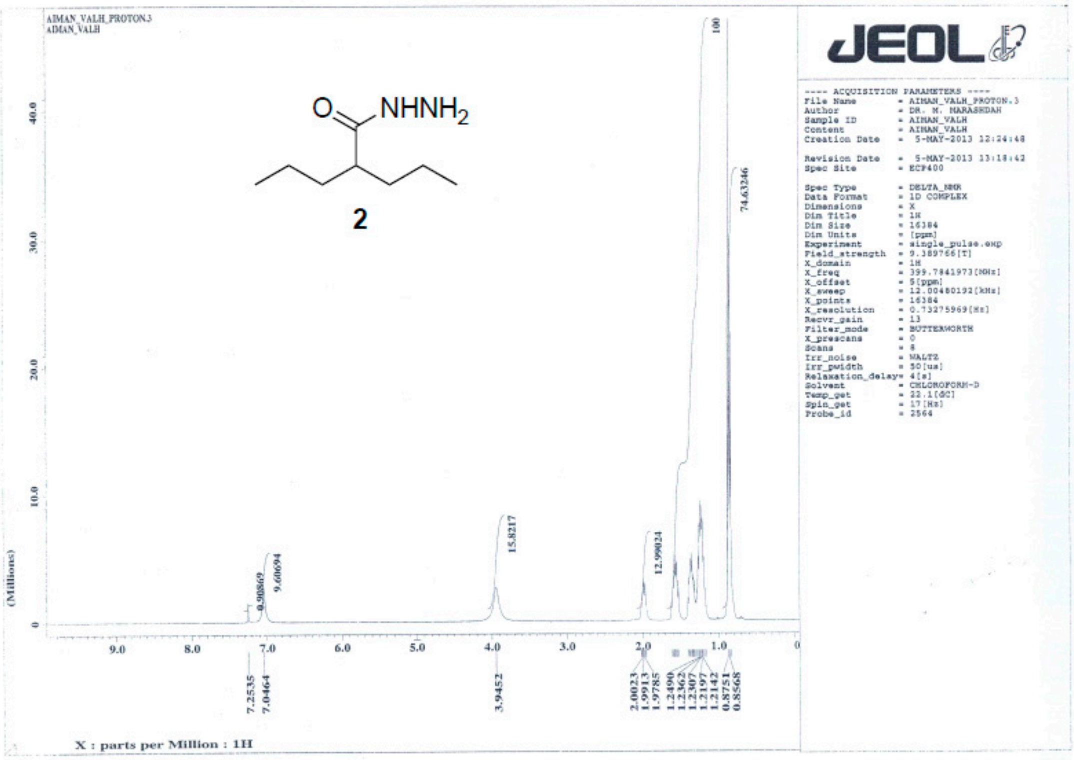


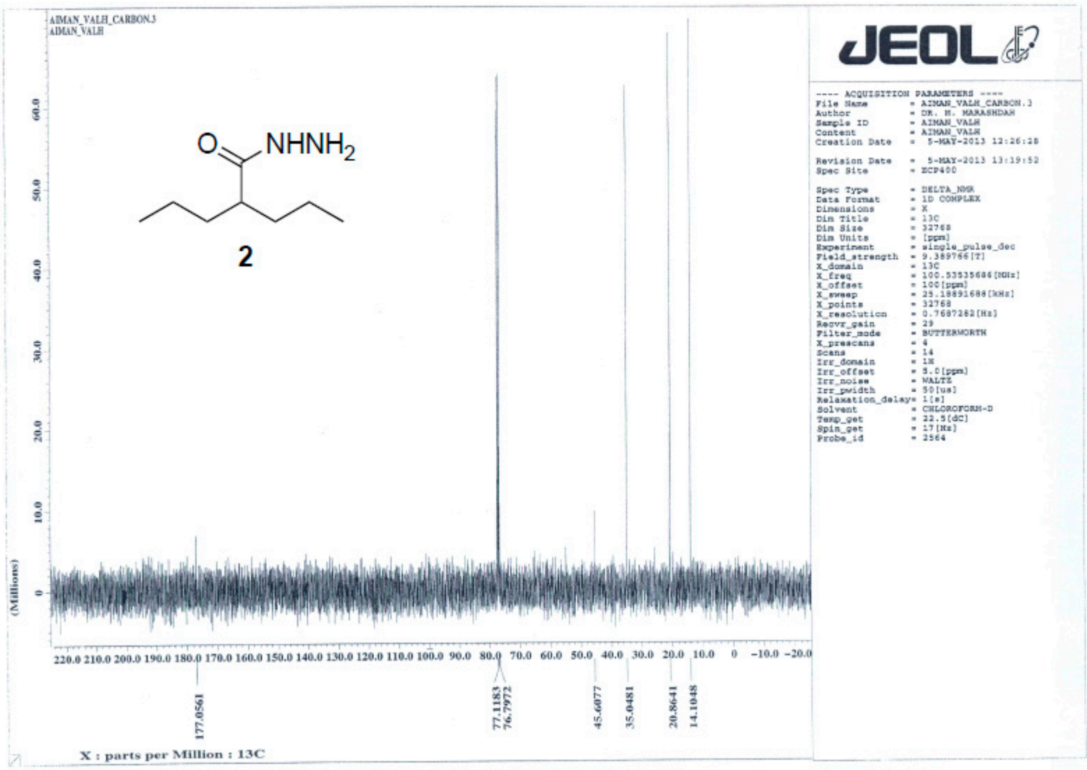


**Figure S1.** $^{1}$H-NMR and $^{13}$C-NMR of Compound **2**.

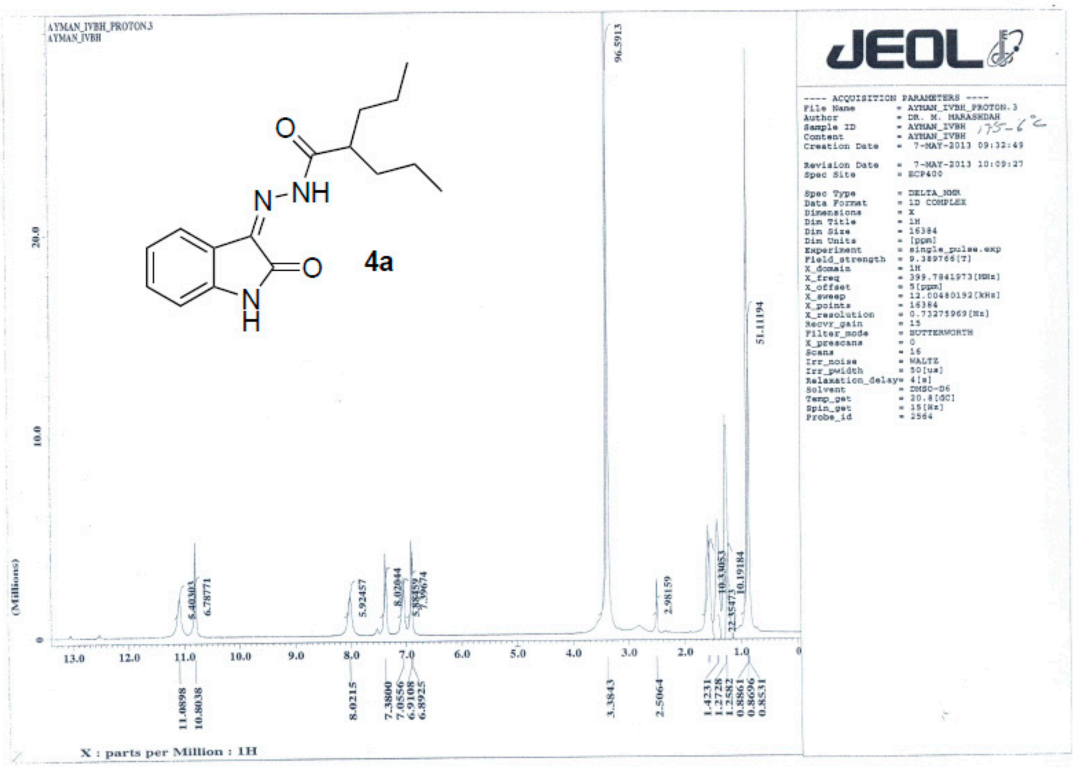

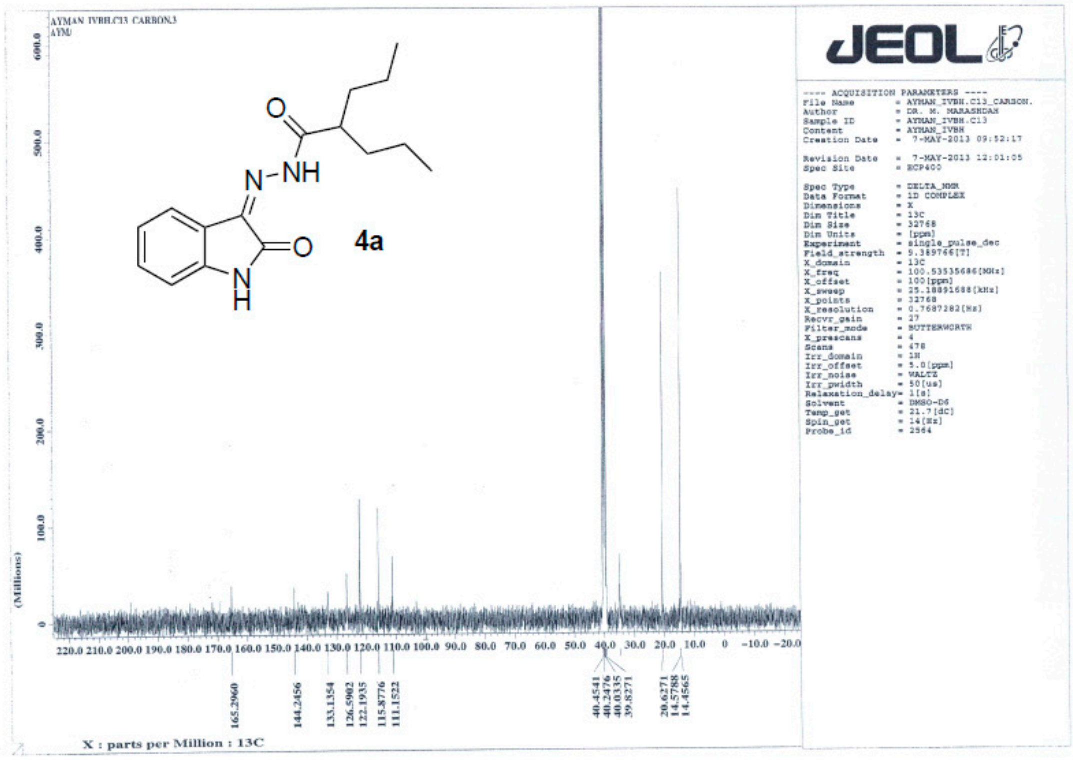

**Figure S2.** $^{1}$H-NMR and $^{13}$C-NMR of Compound **4a**.

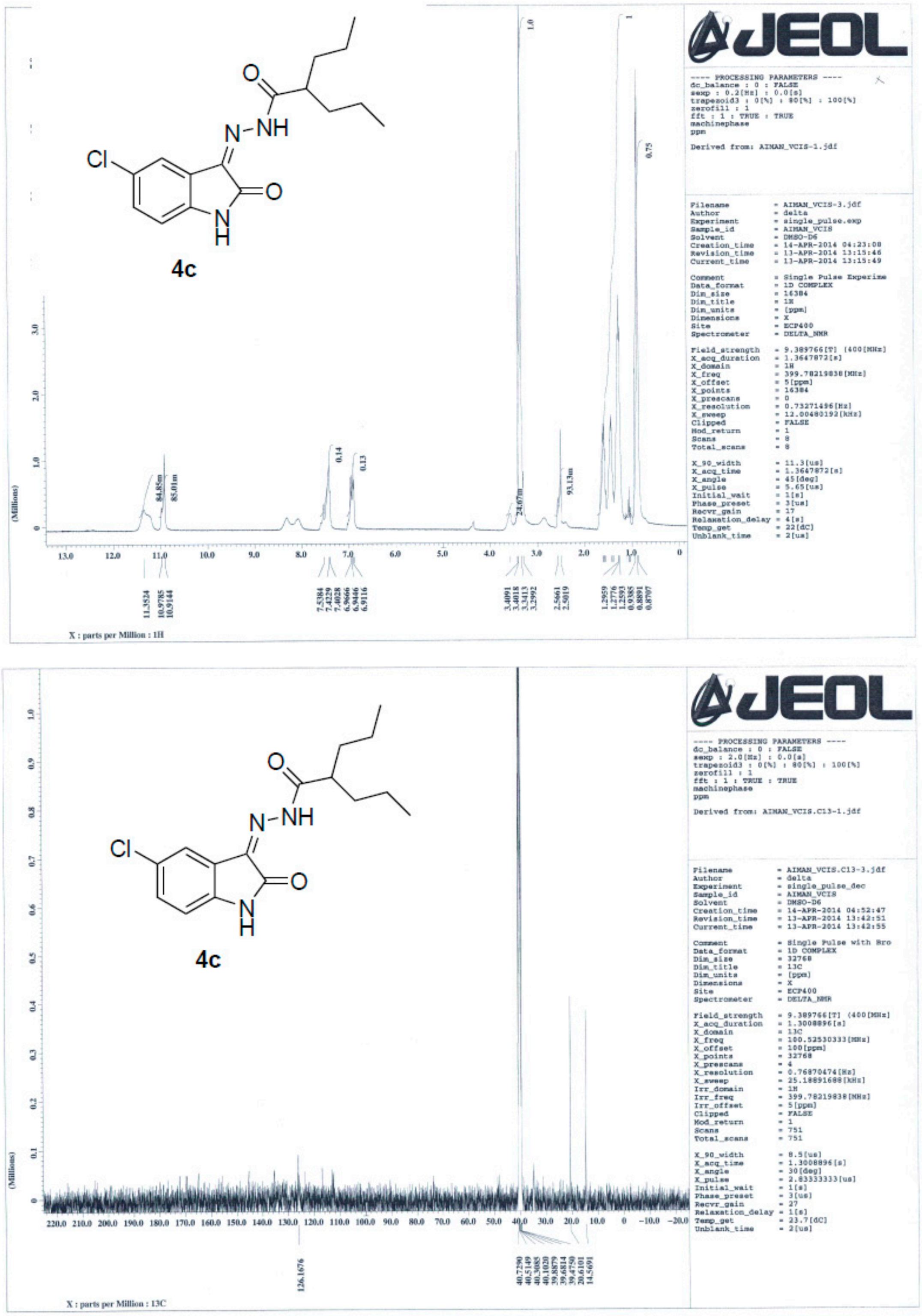


**Figure S3.** $^{1}$H-NMR and $^{13}$C-NMR of Compound **4c**.

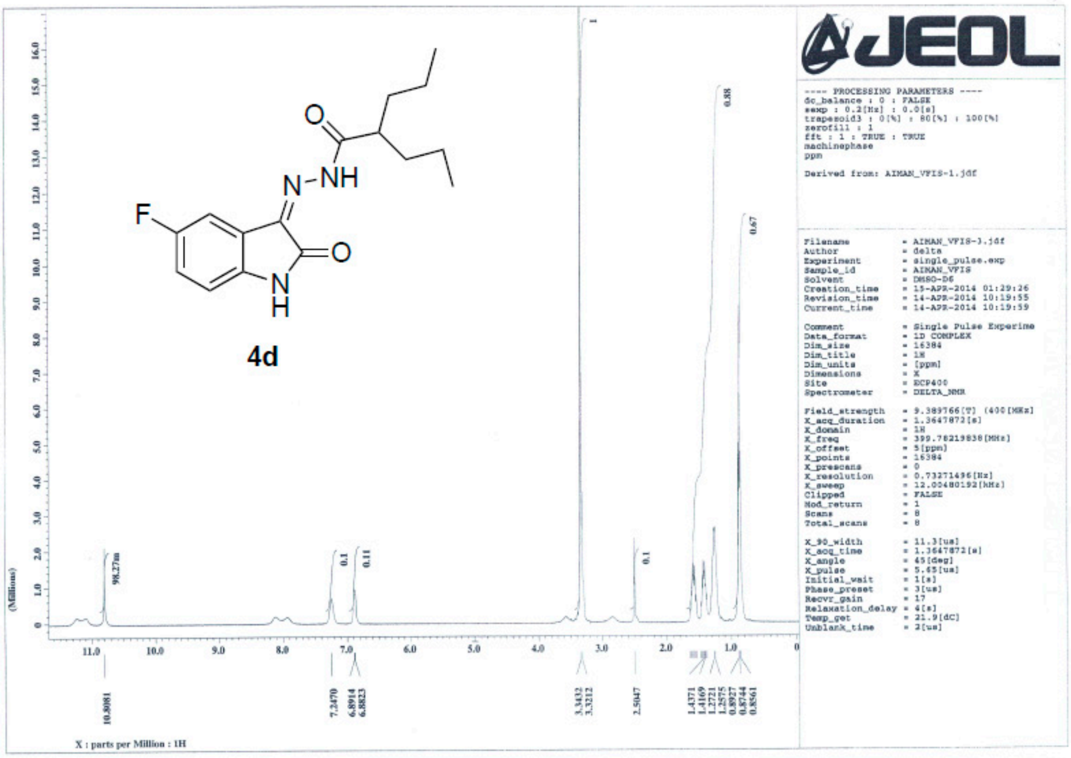


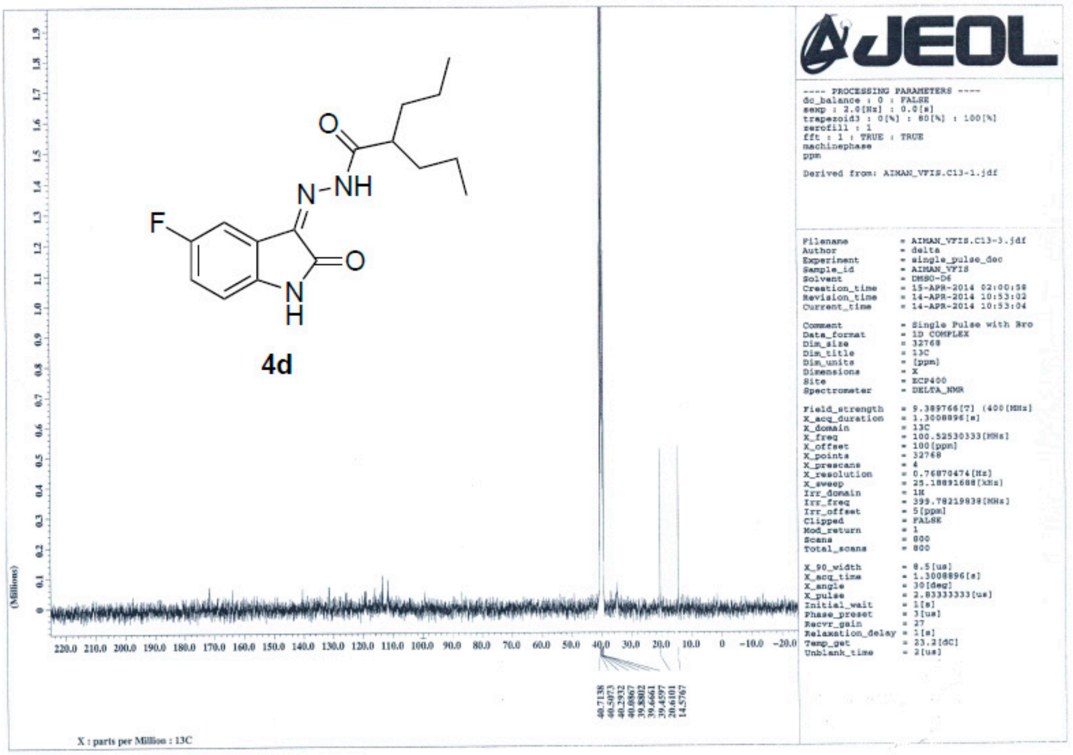


**Figure S4.** $^{1}$H-NMR and $^{13}$C-NMR of Compound **4d**.

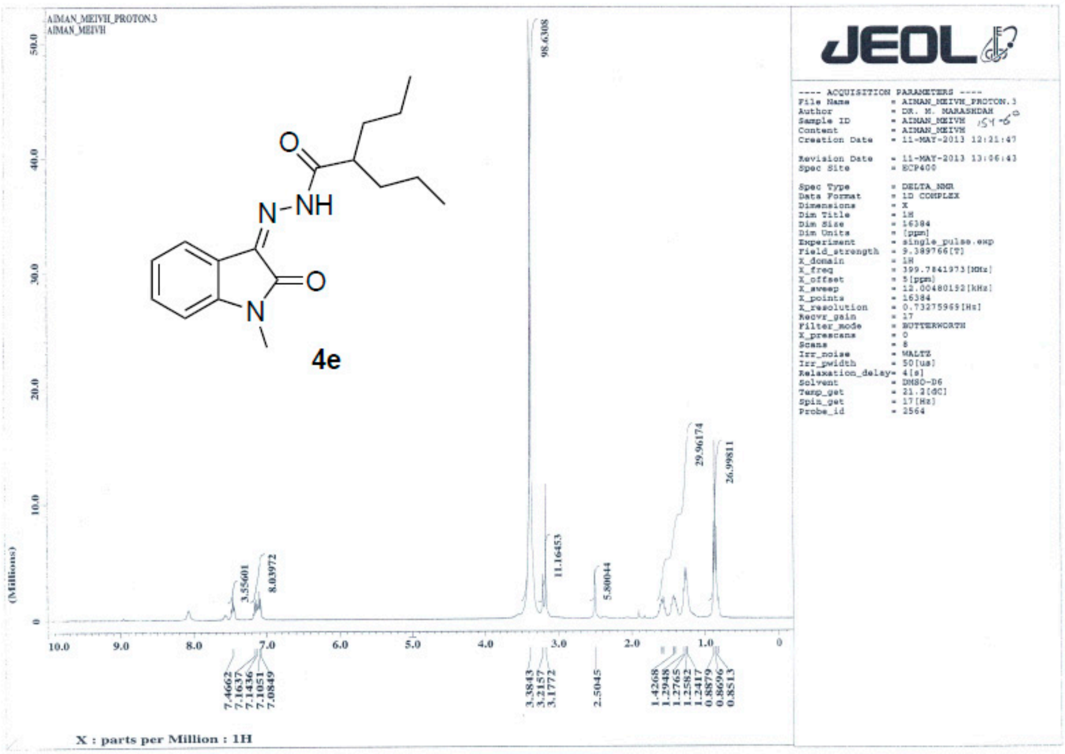

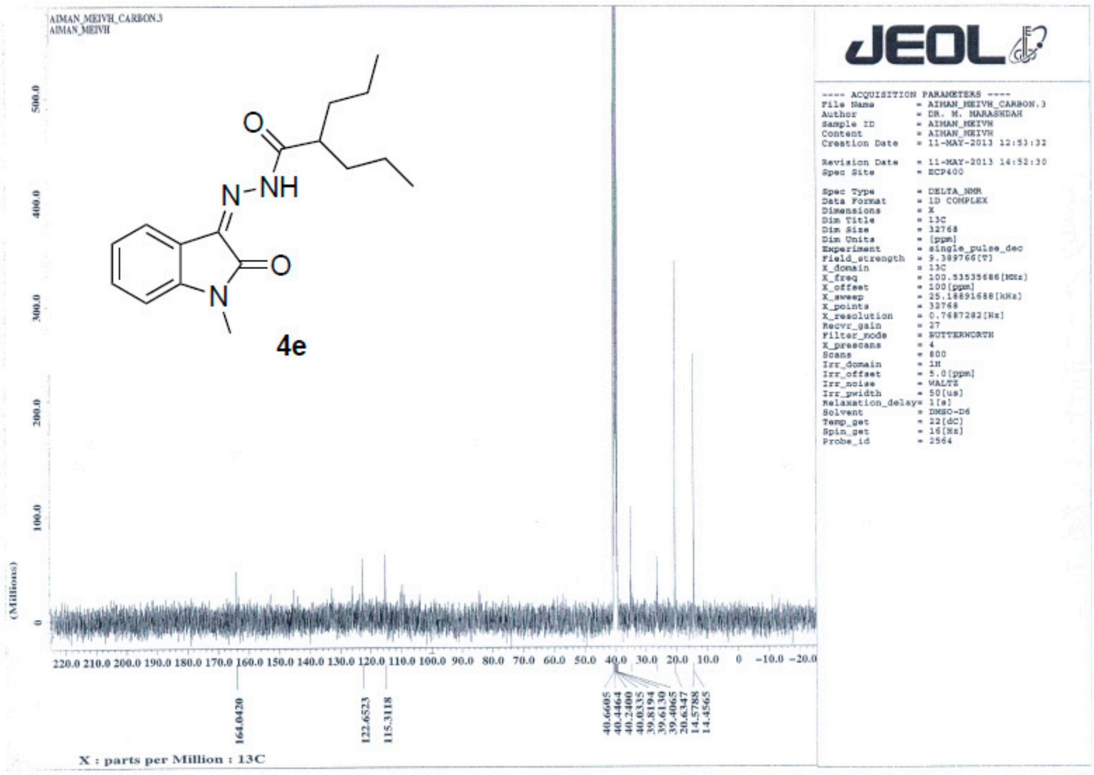

**Figure S5.** $^{1}$H-NMR and $^{13}$C-NMR of Compound **4e**.

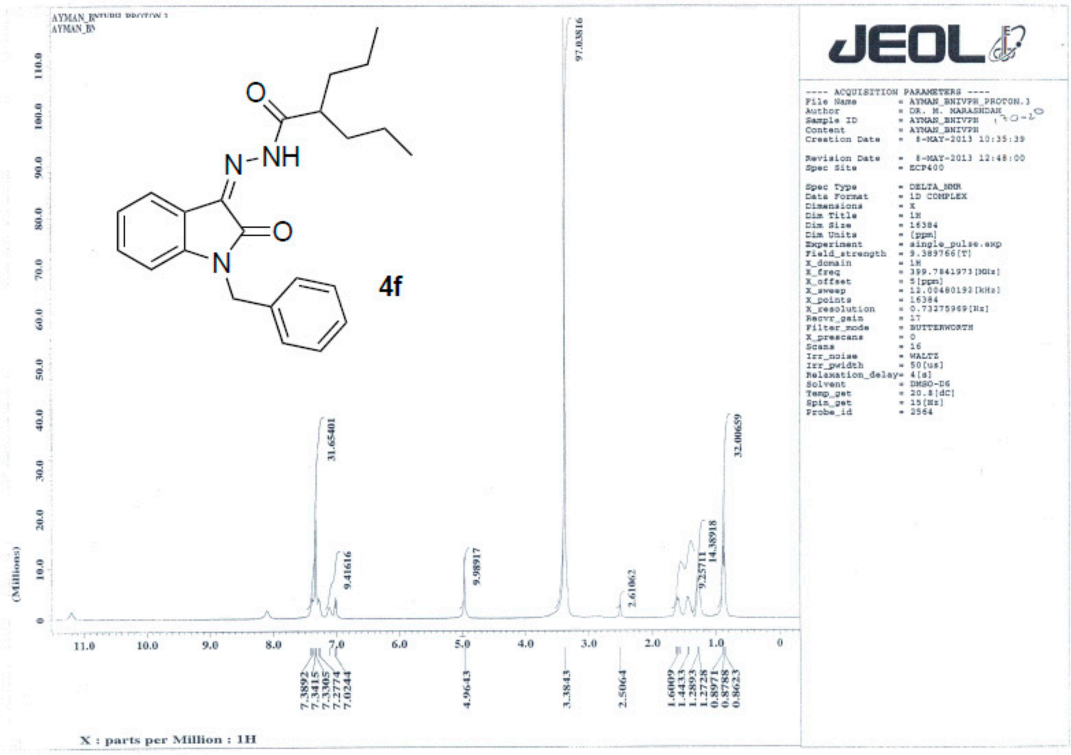

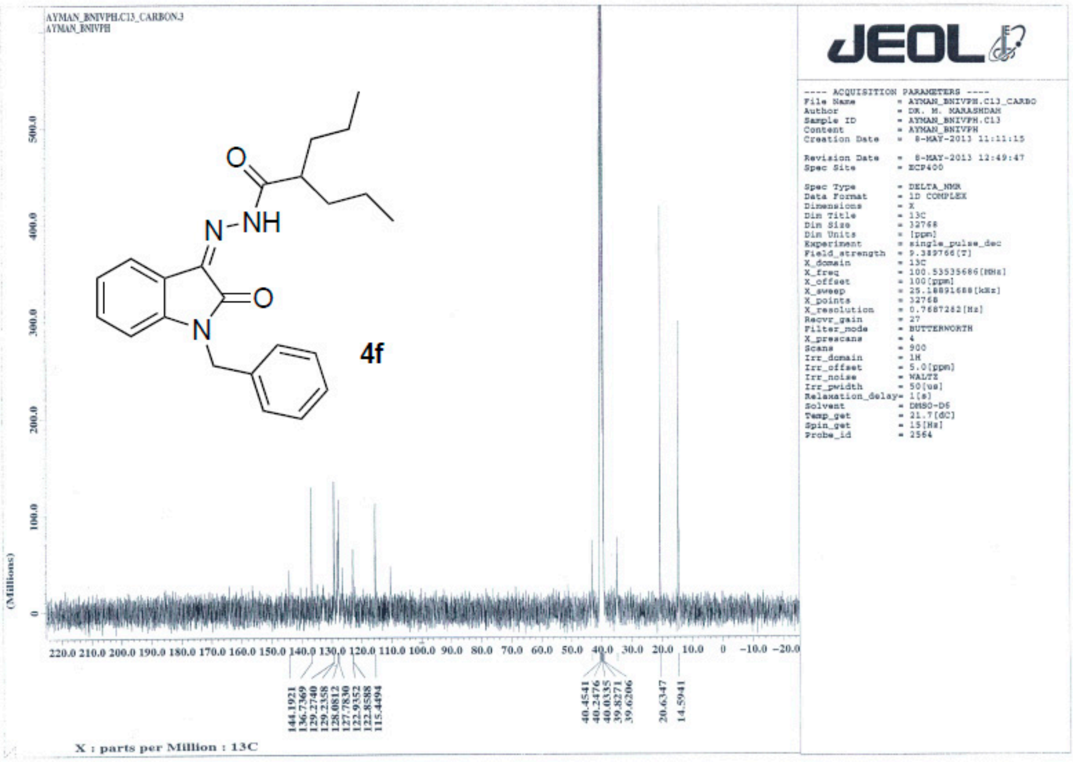

**Figure S6.** $^{1}$H-NMR and $^{13}$C-NMR of Compound **4f**.

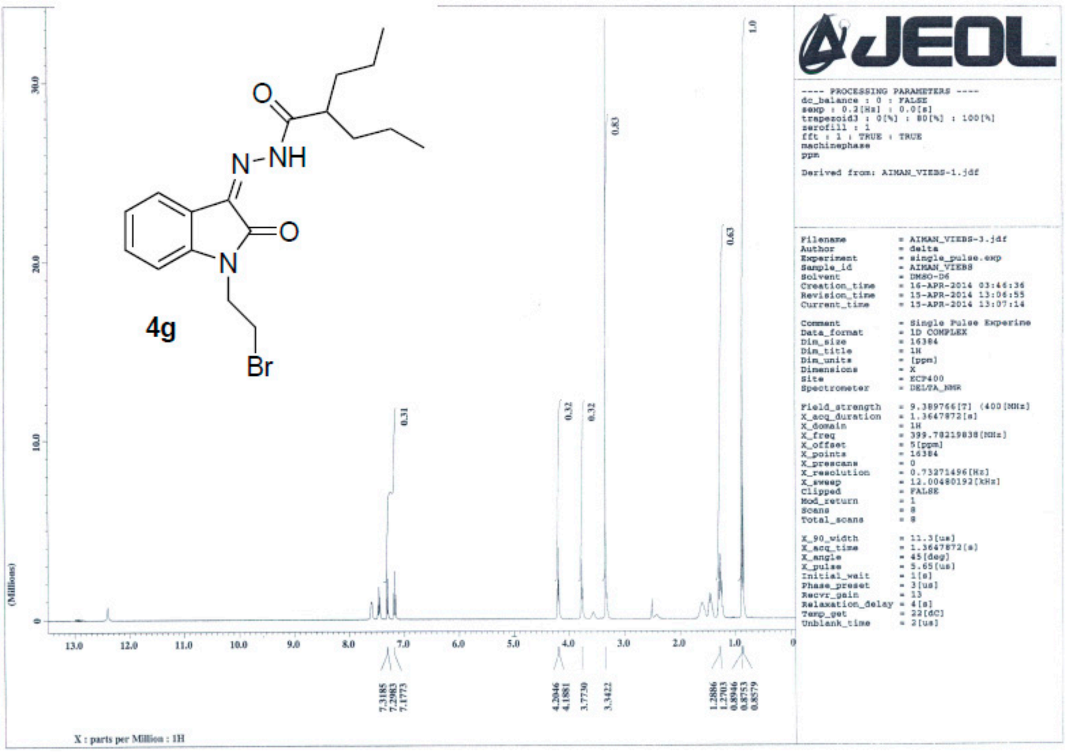


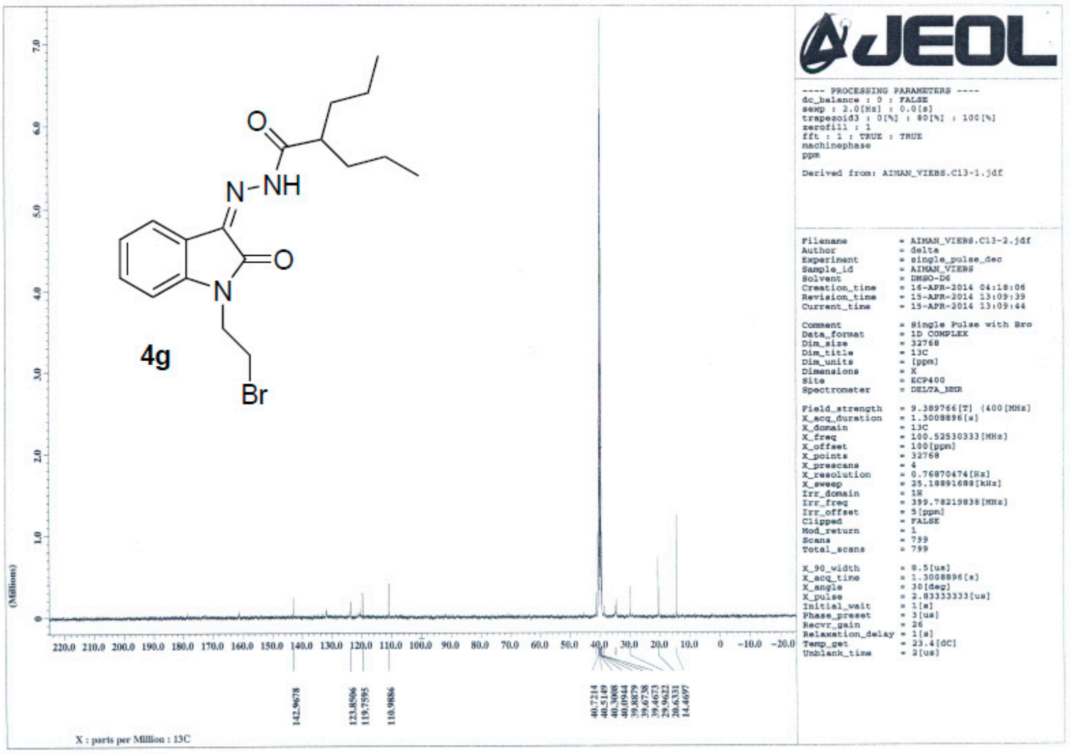


**Figure S7.** $^{1}$H-NMR and $^{13}$C-NMR of Compound **4g**.

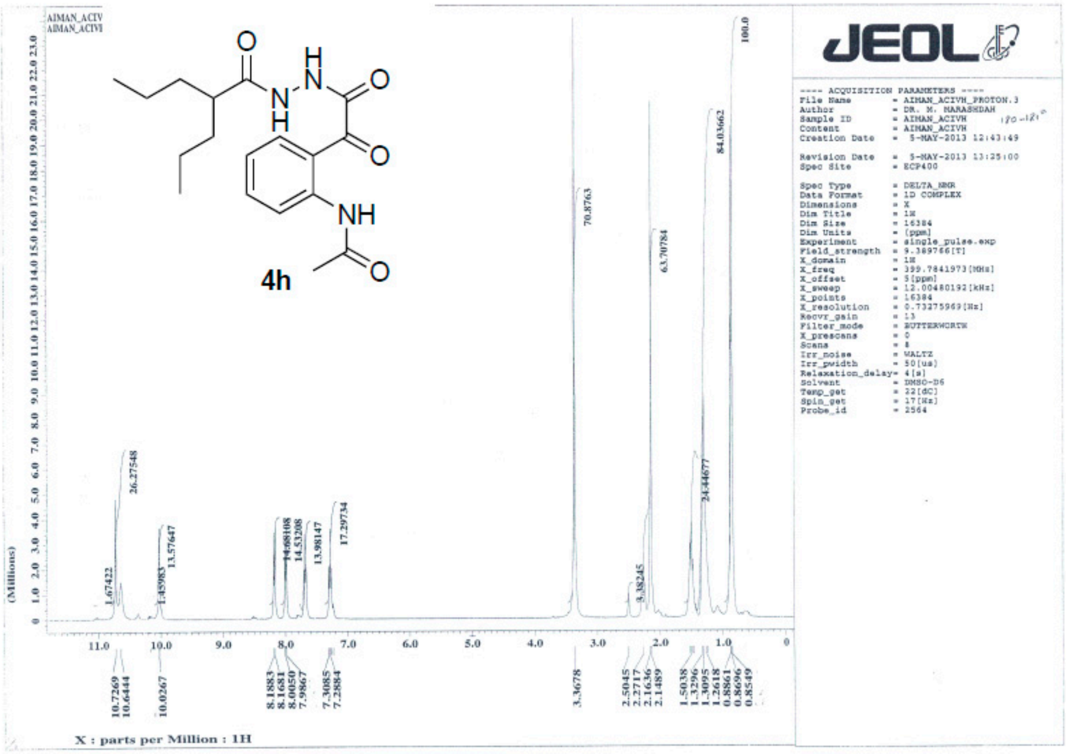


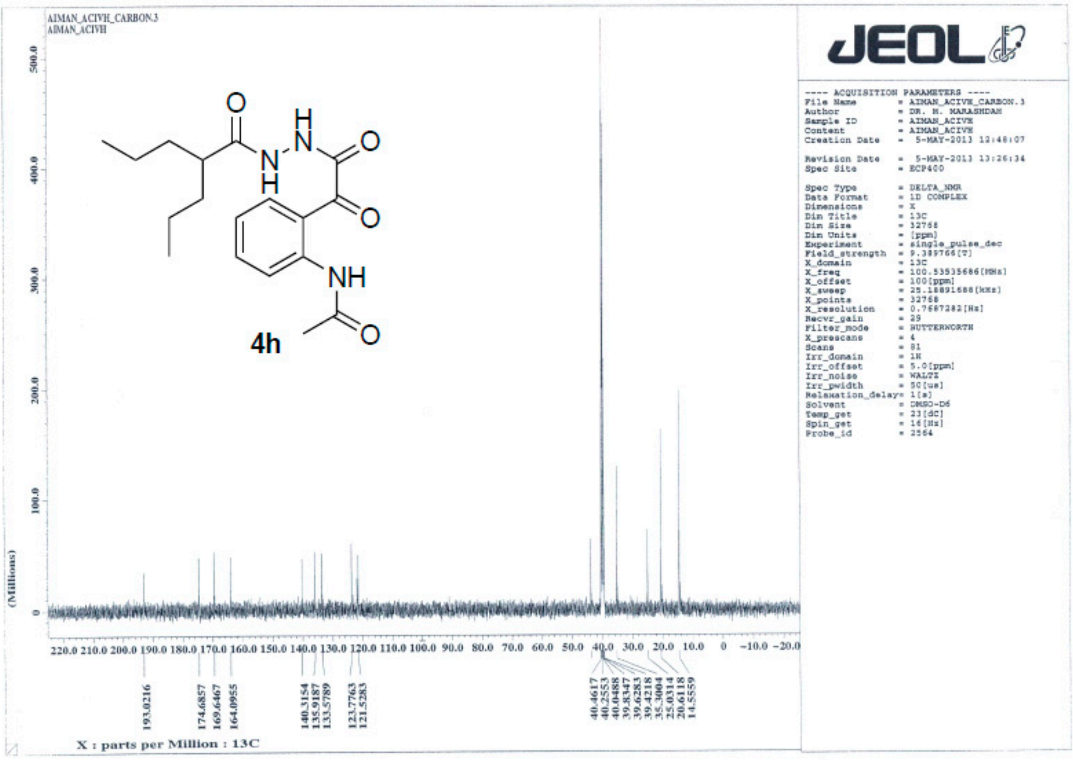


**Figure S8.** $^{1}$H-NMR and $^{13}$C-NMR of Compound **4h**.
